# The deletion of glucagon-like peptide-1 receptors expressing neurons in the dorsomedial hypothalamic nucleus disrupts the diurnal feeding pattern and induces hyperphagia and obesity

**DOI:** 10.1186/s12986-021-00582-z

**Published:** 2021-06-07

**Authors:** Yuko Maejima, Shoko Yokota, Masaru Shimizu, Shoichiro Horita, Daisuke Kobayashi, Akihiro Hazama, Kenju Shimomura

**Affiliations:** 1 Department of Bioregulation and Pharmacological Medicine, Fukushima Medical University School of Medicine, Fukushima, 960-1295 Japan; 2 Department of Cellular and Integrative Physiology, Fukushima University School of Medicine, Fukushima, 960-1295 Japan

**Keywords:** Glucagon-like peptide-1, Glucagon-like peptide-1 receptor, Suprachiasmatic nucleus, Dorsomedial hypothalamic nucleus, Saporin

## Abstract

**Background:**

Feeding rhythm disruption contributes to the development of obesity. The receptors of glucagon-like peptide-1 (GLP-1) are distributed in the wide regions of the brain. Among these regions, GLP-1 receptors (GLP-1R) are expressed in the dorsomedial hypothalamic nucleus (DMH) which are known to be associated with thermogenesis and circadian rhythm development. However, the physiological roles of GLP-1R expressing neurons in the DMH remain elusive.

**Methods:**

To examine the physiological role of GLP-1R expressing neurons in the DMH, saporin-conjugated exenatide4 was injected into rat brain DMH to delete GLP-1R-positive neurons. Subsequently, locomotor activity, diurnal feeding pattern, amount of food intake and body weight were measured.

**Results:**

This deletion of GLP-1R-positive neurons in the DMH induced hyperphagia, the disruption of diurnal feeding pattern, and obesity. The deletion of GLP-1R expressing neurons also reduced glutamic acid decarboxylase 67 and cholecystokinin A receptor mRNA levels in the DMH. Also, it reduced the c-fos expression after refeeding in the suprachiasmatic nucleus (SCN). Thirty percent of DMH neurons projecting to the SCN expressed GLP-1R. Functionally, refeeding after fasting induced c-fos expression in the SCN projecting neurons in the DMH. As for the projection to the DMH, neurons in the nucleus tractus solitarius (NTS) were found to be projecting to the DMH, with 33% of those neurons being GLP-1-positive. Refeeding induced c-fos expression in the DMH projecting neurons in the NTS.

**Conclusion:**

These findings suggest that GLP-1R expressing neurons in the DMH may mediate feeding termination. In addition, this meal signal may be transmitted to SCN neurons and change the neural activities.

**Supplementary Information:**

The online version contains supplementary material available at 10.1186/s12986-021-00582-z.

## Background

“Light exposure” and “meal consumption” signals are two dominant synchronizers of the mammalian circadian clock [1, 2]. The suprachiasmatic nucleus (SCN) is the central biological clock that coordinates behavioral (sleep–wake cycle, feeding etc.) and physiological (thermogenesis, blood pressure etc.) circadian rhythms [3, 4].

The input of information to the brain reflecting the peripheral status after eating and digesting is considered to be through the arcuate nucleus (ARC) and nucleus tractus solitarius (NTS), which are collectively known as the first-order center in food intake regulation [5]. The ARC receives prandial hormone information, which is induced by leptin, ghrelin and insulin [5], while the NTS receives neural prandial information induced by ghrelin, cholecystokinin (CCK), and glucagon-like peptide-1 (GLP-1) via the vagal afferent pathway [5]. ARC neurons are reported to maintain leptin signal-related feeding rhythms independent of light entrainment [6]. However, to our knowledge, there have been no reports to date concerning the NTS and meal-induced signals for SCN. In the present study, we investigated whether meal-induced signals via the vagal afferent to NTS may influence the activity of SCN neurons.

Neurons in the NTS contain various neuropeptides, such as proopiomelanocortin (POMC), cocaine- and amphetamine-regulated transcript (CART), prolactin-releasing peptide (PrRP), and GLP-1 [7].

GLP-1 is derived from preproglucagon (PPG) and is widely known as an anorexigenic peptide and its analogue has already been approved as an anti-obesity drug by the FDA [8]. GLP-1 receptors (GLP-1R) are distributed throughout various regions of the central nervous system [9]. PPG neurons are distributed in hypoglossal nucleus and reticular nucleus, as well as NTS [10]. These PPG neurons are projected to the various brain regions, such as DMH, paraventricular nucleus (PVN), ventromedial hypothalamic nucleus (VMH), arcuate nucleus (ARC), paraventricular thalamic nucleus (PVT), periaqueductal gray (PAG) and bed nucleus of the stria terminalis (BNST) [10, 11]. The chemogenetic stimulation of PPG neurons in the NTS and ventrolateral medulla reduces metabolic rate and food intake in fed and fasted state [12]. The knocking down of PPG in NTS by shRNA induces hyperphagia and body weight (BW) gain [13]. On the other hand, ablation of PPG neuron in the NTS or acute chemogenetic inhibition of PPG neurons does not affect ad libitum feeding, but increased refeeding intake after fasting [14]. Thus, endogenous GLP-1 in the central nervous system may play an important role in physiological food intake regulation [15].

The DMH is one of the important components of the hypothalamus, regulating food intake and the autonomic nervous system and receive abundant projection from PPG neurons [10]. Various neuron species, such as neuropeptide Y (NPY) [16], CART [17] and gamma-aminobutyric acid (GABA) neurons are distributed in the DMH [18]. DMH neurons are known to abundantly express GLP-1R [19]. Knocking down of GLP-1R in DMH increased BW with reduced thermogenesis in brown adipose tissue and increased adiposity but with no effect on the daily food intake [19]. However, DMH lesions exhibit a lower amplitude and mean level of light–dark entrained activity [20]. It has been known that the DMH plays an important role as a central food-entrainable oscillator in the feeding mediated regulation of circadian behavior [21].

Therefore, we investigated whether meal-induced signals via the NTS may give influence on activity of SCN neurons via NTS GLP-1 and DMH GLP-1R expressing neurons.

In the present study, we report a possible neural pathway that integrates meal-evoked information from the NTS to the SCN with a GLP-1R expressing neurons in the DMH acting as a modifying factor.

## Methods

### Animals and housing

Male Wistar rats (9 weeks old) were purchased from Japan SLC. The animals were maintained on a 12-h light/dark cycle and given conventional food (CE-2; Clea, Osaka, Japan) and water ad libitum. The light was turned on at 7:00 AM, with zeitgeber time (ZT) 0, meaning onset of the light phase. In all the experiments measuring the food intake and body weight in more than two groups, initial body weight of different groups were matched prior to the start to the experiments. Experimental procedures and care of animals were carried with the approval of the Fukushima Medical University Institute of Animal Care and Use Committee.

### Immunostaining of c-Fos

Food was deprived at ZT10 on the day before the experiment. After 16 h of fasting, chow was given at ZT2. After 2 h of refeeding, the rats were injected with a mixture of three types of anaesthetic agents [(0.003%, Domitor, Nippon Zenyaku Kogyo Co., Ltd., Koriyama, Japan), midazolam (0.04%, Dormicum, Astellas Pharma Inc., Tokyo, Japan), and butorphanol tartrate (0.05%, Vetorphale, Meiji Seika Pharma Co., Ltd., Tokyo, Japan)] (5 ml/kg) and perfused intracardially with 4% paraformaldehyde (PFA), and 0.2% picric acid.

Serial coronal sections (40 μm) were collected from each rat using a freezing microtome. The sections were then prepared from 0.48 mm to -14.7 mm from the bregma, washed in PBS (0.01 M, pH7.4), then incubated for 20 min with 0.3% H_2_O_2_. Next, the sections were incubated for 1 h in a blocking solution comprising of 0.3% TritonX-100, 2% bovine serum albumin (BSA), and 2% normal goat serum (NGS) and then incubated with rabbit anti-c-Fos antibody (sc-52, 1: 1000, Santa Cruz, CA) in the blocking solution overnight at 4 °C. Next, they were incubated with biotinylated goat anti-rabbit IgG (diluted to 1:500, Vector Laboratories, CA) for 30 min, and were then incubated with an avidin–biotin-peroxidase complex (Vectastain Elite ABC Kit; Vector Laboratories, CA) for 60 min. Immunoreactions were visualized by incubation in a 0.02% diaminobenzidine solution containing 0.3% nickel ammonium and 0.015% H_2_O_2_ for 5 min. After color development, the sections were mounted on glass slides and covered.

### Immunostaining of GLP-1R

For GLP-1R staining, DMH-containing sections were incubated with the blocking solution (0.1% Triton-X, 2% BSA, 5% NGS) for 30 min. These sections were then incubated with a rabbit anti-GLP-1R antibody (1:200, ThermoFisher, MA) overnight at 4 °C. Then, the sections were incubated with Alexa 488-labelled anti-rabbit IgG (Invitrogen, CA) for 30 min and mounted with a DAPI-containing mount medium on glass slides and covered with a cover glass. The images were acquired using a confocal laser-scanning microscope (Fluoreview FV10i; Olympus, Osaka, Japan). To examine the specificity of the antibody for GLP-1R, absorption test and western blotting were performed (Additional file 1: Figs. S1 and S2).

**Fig. 1:**
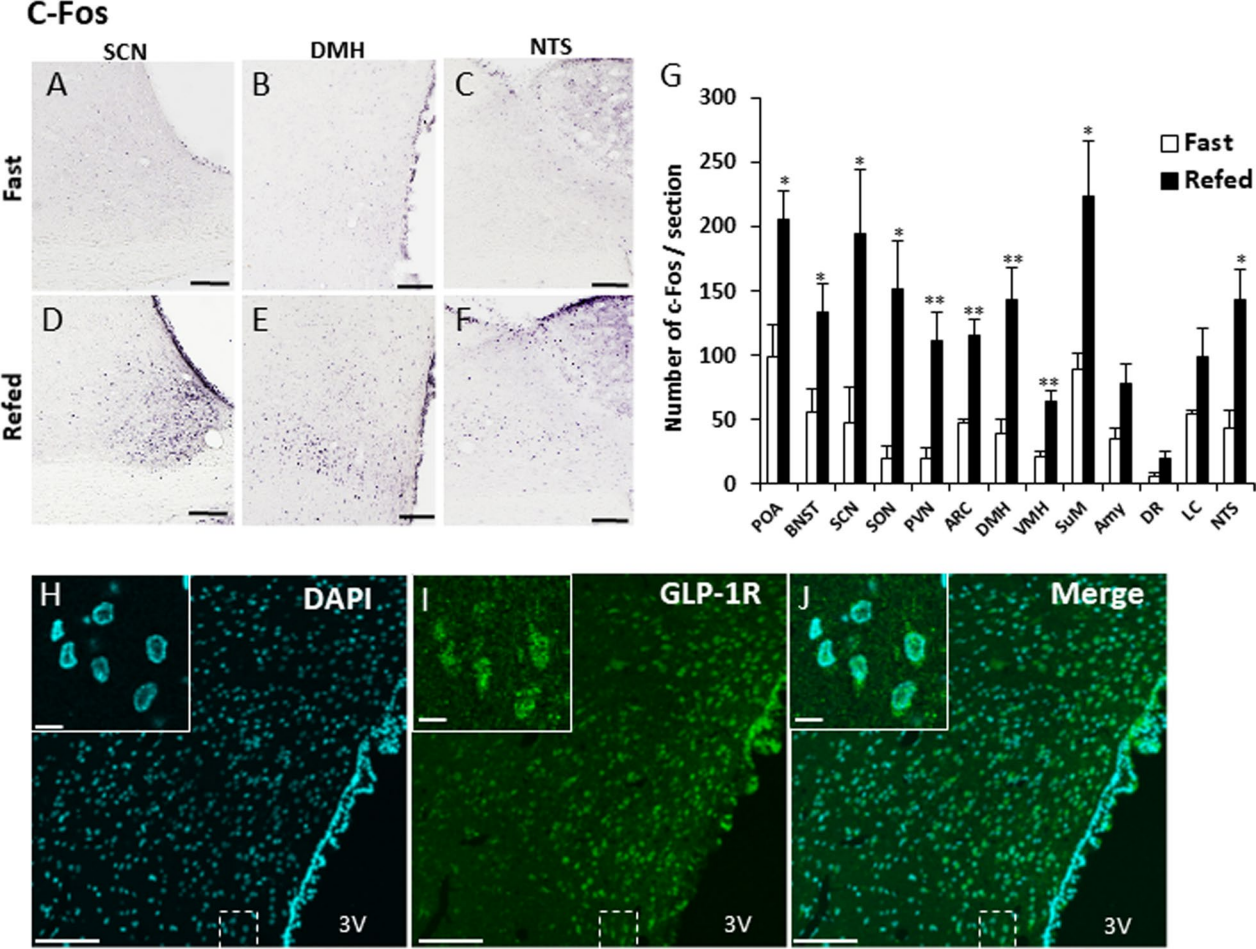
The c-Fos expression in the brain after refeeding and distribution of GLP-1R in the DMH. **A**–**F**: The representative images of c-Fos expression in the SCN (**A**, **D**), DMH (**B**, **E**) and NTS (**C**, **F**) after fasting (**A**–**C**) or refeeding (**D**–**F**) rats. Refeeding was performed for 2 h after 16 h of fasting in normal 12 h light/12 h dark conditions. Scale bar = 100 μm. G: The number of c-Fos per sections in feeding-related brain regions. (*n* = 4 each). ARC: arcuate nucleus, Amy: amygdala, BNST: bed nucleus of stria terminalis, DMH: dorsomedial hypothalamic nucleus, DR: dorsal raphe, LC: locus coeruleus, NTS: nucleus tractus solitarius, SCN: suprachiasmatic nucleus, SON: supraoptic nucleus, SuM: supramammillary nucleus, POA: preoptic area, PVN: paraventricular hypothalamic nucleus, VMH: ventromedial hypothalamic nucleus. **H**–**J** DAPI nuclear staining (**H**) and GLP-1R immunostaining (**I**) in the DMH and a merged image of **H** and **I** (**J**). Scale bars = 100 μm. Each image located in the upper left corner of **H**–**J** is an enlarged image of the dashed square. Scale bars = 10 μm

**Fig. 2:**
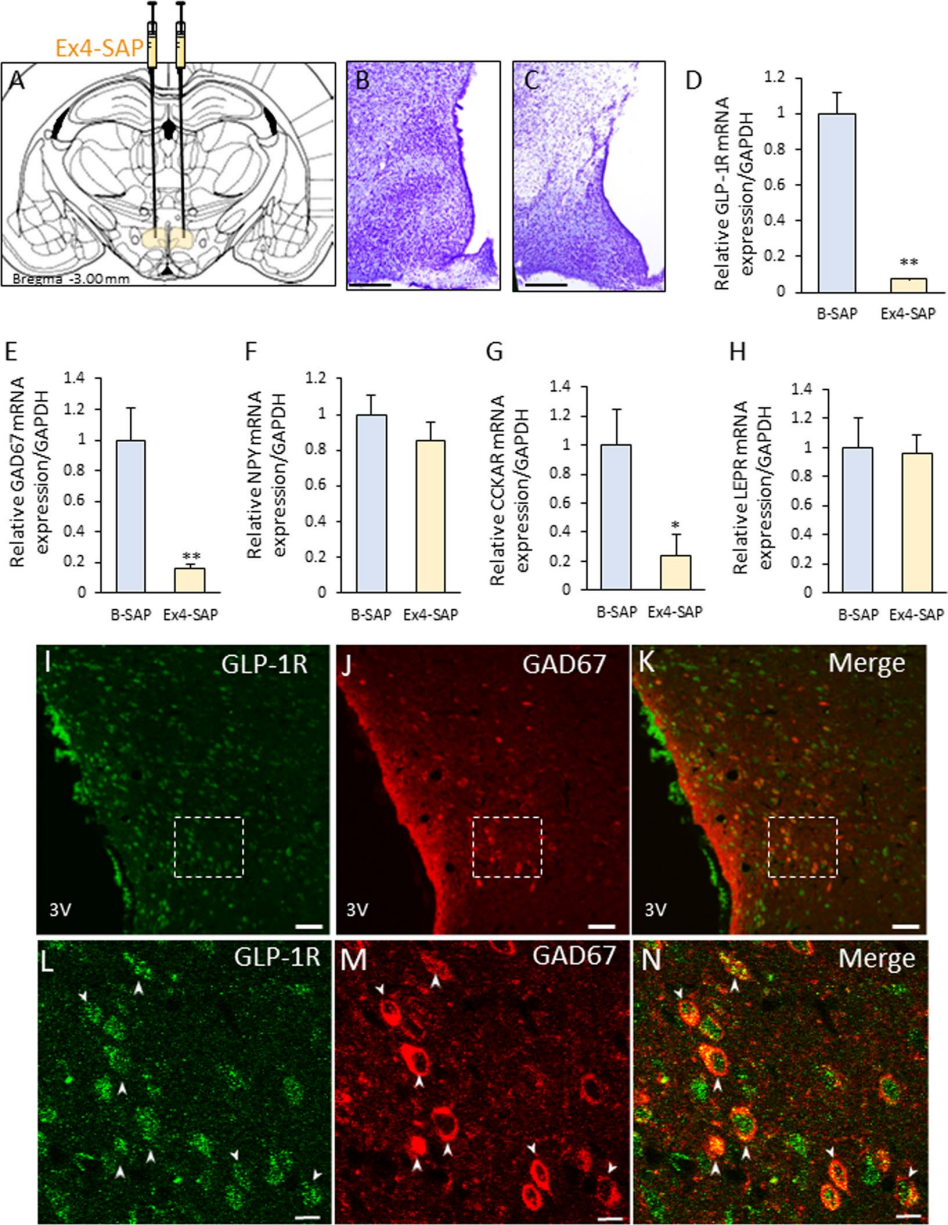

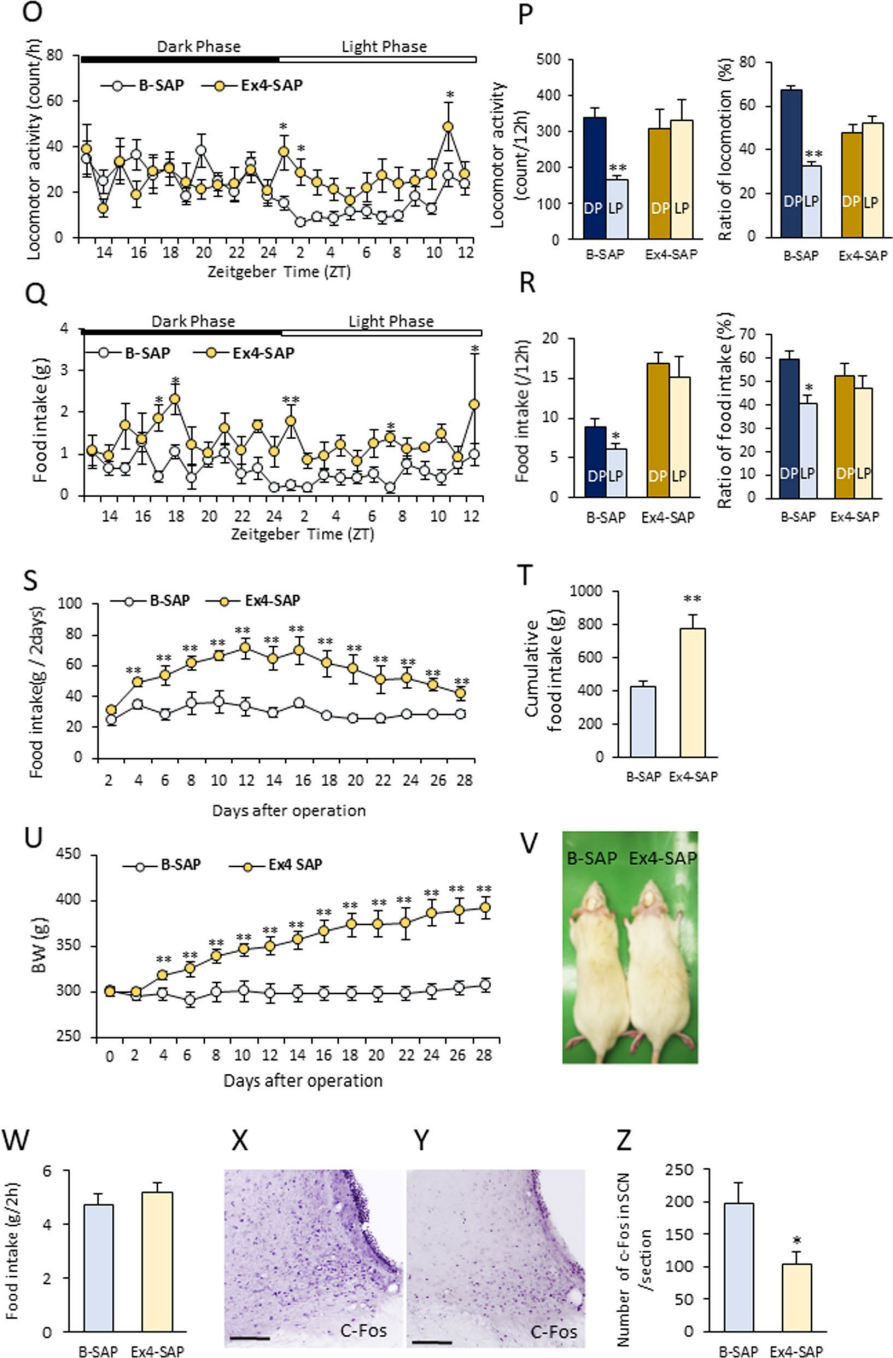
Deletion of GLP-1R expressing neurons in the DMH results in hyperphagia and obesity. **A**–**C**: An illustration of the control blank-saporin (B-SAP) or Exenatide4-saporin (Ex4-SAP) injection site (**A**). The sections with Nissl staining after B-SAP (**B**) or Ex4-SAP (**C**) injection. **D**–**H** Relative mRNA expression of GLP-1R (**D**), GAD67 (**E**), NPY (**F**), CCKA receptors (CCKAR) (**G**) and leptin receptors (LEPR) (**H**). **I**–**N** Colocalization of GLP-1R (**I**, **L**) and GAD67 (**J**, **M**) in the DMH. **K** and **N** indicate the merged image of **I** and **J**, **L** and **M**, respectively. **l**–**N** indicate enlarged image of dashed square in **I**–**K**, respectively. Scales in **I**–**K** = 50 μm. Scales in **L**–**N** = 10 μm. **O** Twenty-four hours of locomotor activity (count per hour) of rats injected with control B-SAP or Ex4-SAP into the bilateral DMH. (n = 9, 7) **P** The counts of locomotor activity (Left), and the ratio of locomotor activity (Right) between the dark phase (DP) and light phase (LP) in B-SAP- and Ex4-SAP-injected rats. * *P* < 0.05, ** *P* < 0.01. Student *t*-test. (n = 9, 7). **Q** Twenty-four hours of food intake in rats that were injected with B-SAP or Ex4-SAP. *P < 0.05, **P < 0.01. Two-way ANOVA. (n = 9, 6). **R** The amount of food intake (Left) and the ratio of food intake (Right) between the dark phase (DP) and light phase (LP) in the B-SAP- and Ex4-SAP-injected rats. * *P* < 0.05, ** *P* < 0.01. Student’s *t*-test. (n = 9, 6). S: Daily food intake for 4 weeks after B-SAP or Ex4-SAP injection into the DMH. *P < 0.05, **P < 0.01. Two-way ANOVA. (n = 6, 6) **T** Cumulative food intake for 4 weeks in the B-SAP- and Ex4-SAP-injected rats. *P < 0.05. Student’s *t*-test. (n = 6, 6). **U** Change of BW for 4 weeks after B-SAP or Ex4-SAP injection. ** *P* < 0.01. Two-way ANOVA. (n = 6, 6). **V** The comparison of body size of the B-SAP- and Ex4-SAP-injected rats at 28 days after injection. Left: B-SAP, Right: Ex4-SAP. W: Food intake for 2 h after refeeding in B-SAP or Ex4-SAP injected rats. (n = 9, 8). **X**, **Y** Representative c-Fos expression after refeeding in the SCN of rats in which B-SAP (**X**) or Ex4-SAP (**Y**) was injected into the DMH. Scale bars = 100 μm. **Z** The number of c-Fos-positive neurons in the SCN per section after being refed. * P < 0.05, Student’s *t*-test. (n = 9, 8)

### The injection of Exenatide 4-saporin into DMH

All animals were isolated in individual cage and habituated for 10 days before operation. Exenatide 4-saporin (Ex4-SAP), which internalize upon binding to GLP-1 receptor and induce cell death by disrupting ribosomal function [22], was purchased from the Advanced Targeting System (CA), and blank saporin (B-SAP) was used for control injections.

Injections of 0.1 μg/0.5 μl Ex4-SAP or 0.1 μg/0.5 μl B-SAP were administered into the DMH 3.0 mm caudal to the bregma, 0.5 mm lateral from the midline, and 8.6 mm below the surface of the skull; injection speed: 0.1 μl/min) by using modified glass pipettes under anaesthesia. To prevent the B-SAP or Ex4-SAP solution from adhering to the outside of the target area, the tip of the glass pipette was fixed to the injection site for 10 min after injection. BW and food intake of the animals injected with B-SAP or Ex4-SAP were measured at ZT10 for 28 consecutive days.

At 10 days after operation, rats were left inside the chamber of activity monitoring system (ACTIMO-100: Shin factory, Fukuoka, Japan) combined with food intake measurement apparatus. During the habituation period, food intake and BW were also measured at ZT10. After 4 days of habituation, hourly locomotor activity and food intake were measured for 24 h. Locomotor activity was estimated by the number of inhibitions of infrared beams in both X and Y directions. After the measurement, rats were returned to individual home cage, and measurement of food intake and BW were continued. At 29 days after injection, food was deprived at ZT10. Then, after 16 h of fasting, food was administered to the animals at ZT2, and food intake was measured for 2 h. The animals were then perfused and their brains were removed.

Brain sections were applied with Nissl and c-Fos staining. C-Fos staining in SCN containing sections and the counting of c-Fos positive neurons were performed in the same manner as described above. C-Fos positive neurons are counted under light microscope.

### Quantitative real-time PCR

B-SAP or Ex4-SAP was injected into bilateral DMH, as described above. Thirty days after B-SAP or Ex4-SAP injection, animals were deeply anesthesized by a mixture of three types of anesthetic agents (5 ml/kg), and removed brain. DMH containing brain slice (Bregma from -2.6-3.3) was sliced by using brain matrix and manually punched out DMH (Additional file 1: Fig. S 2). Total RNA was isolated using a RNeasy minikit (QIAGEN, Hilden, Germany) and Monarch RNA Purification Culumns (New England BioLabs Japan, Inc., Massachusetts, USA). c-DNA synthesis was performed using a M-MLV (Thermo Fisher Scientific, Massachusetts, USA), RNaseOUT Recombinant Ribonuclease Inhibitor (Thermo Fisher Scientific, Massachusetts, USA), and dNTP (Agilent Technologies, Texas, USA). A quantitative RT-PCR assay was performed using the TB Green Premix Ex Taq II (Tli RNaseH Plus, Takara Bio Inc., Shiga, Japan). The cycling condition was as follows: initial denaturation at 95 °C for 30 sec, then 40 cycles at 95 ºC for 5 sec, 56°C for 10 sec, 72°C for 15 sec, according to the protocol. Product accumulation was measured in real time and the mean cycle thresholds were determined. The expression levels of GLP-1R, GAD67, NPY, CCKA receptor (CCKAR) and leptin receptors (LEPR) were calculated using the 2ΔΔCT method of relative quantification and normalized by the housekeeping gene glyceraldehyde-3-phosphate dehydrogenase (GAPDH). The PCR primers are shown in Table 1.

**Table 1:** List of primers used in this study

Name	Primers	
GLP-1 receptor	Fw:GCTGCCCTCAAGTGGATGTA	Rv:ATGAGCAGGAACACCAGTCG
GAD67	Fw:CAAGTTCTGGCTGATGTGGA	Rv:GCCACCCTGTGTAGCTTTTC
NPY	Fw:ATGCTAGGTAACAAACG	Rv:ATGTAGTGTCGCAGAG
CCKA receptor	Fw:AACTCTACCAAGGAATCA	Rv:GTAACAGCCATCACTATC
Leptin receptor	Fw:AGTGGGAAGCACTGTGCAGTT	Rv:GAGCTCTGATGTAGGACGAATAGATG

### Double immunostaining for GLP-1R and GAD67

Non-treated rats were perfused as described above. DMH containing brain sections were incubated for 60 min with blocking solution. These sections were then incubated with rabbit GLP-1R antibody (1:200, Thermofisher, MA) and mouse anti-GAD67 monoclonal antibody (1:500, Merck Millipore, Darmstadt, Germany) overnight at 4 °C. Then, the sections were incubated 40 min with Alexa 488-labelled anti-rabbit IgG and daylight 594-labelled anti-mouse IgG (1:400, Invitrogen, CA). The sections were mounted on glass slides and covered. Confocal fluorescence images were acquired and GLP-1R positive, GAD positive, and double-positive neurons were counted.

### The study of projection to SCN/DMH

A retrograde tracer, cholera toxin subunit B Alexa Fluor 488 (CTB: Invitrogen, CA), was used to identify the projections to the SCN or DMH. CTB, 0.5 μl of 0.5 mg/ml, was acutely injected into the unilateral SCN (1.3 mm caudal to the bregma, 0.3 mm lateral from the midline, and 9.2 mm below the surface of the skull) or the DMH (3.0 mm caudal to the bregma, 0.5 mm lateral from the midline, and 8.6 mm below the surface of the skull) by using modified glass pipettes under anesthesia. The injection speed was 0.5 μl/5 min. To prevent the CTB solution from adhering outside of the target area, the tip of the glass pipette was fixed in the injection site for 10 min after injection. Six days after the administration of CTB, the animals were perfused. The brains injected with CTB into the SCN were used for GLP-1R immunostaining in the DMH, and the brains injected with CTB into the DMH were used for GLP-1 staining in the NTS.

GLP-1R immunostaining in the DMH was performed in the same manner as described above, except for a second antibody. Alexa 594-labelled anti-rabbit IgG (Invitrogen, CA) was used as a second antibody. Brain sections were made using a freezing microtome. Injection accuracy was confirmed histologically.

The animals that were injected with CTB into the DMH were used for GLP-1 immunostaining. Brainstem sections containing the NTS were incubated 60 min with a blocking solution. These sections were then incubated with a monoclonal mouse anti-GLP-1 antibody (1:500, Abcam, Cambridge, UK) overnight at 4 °C. Then, the sections were incubated with Alexa 405-labelled anti-mouse IgG for 30 min, mounted on glass slides and covered with a cover glass. Confocal fluorescence images were acquired. CTB-positive neurons, GLP-1 IR neurons, and double-positive neurons were counted from the fluorescence images.

### Immunostaining of c-Fos after refeeding in the section containing CTB tracer

CTB Alexa Fluor 488 (CTB488: Invitrogen, CA) was injected into the DMH or SCN. The injections were performed in the same manner as described above.

Five days after injection, fasting, refeeding and perfusion were performed in the same manner as described above. The brain sections were prepared from − 2.4 mm to − 14.5 mm from the bregma. The sections were then incubated for 60 min with the blocking solution and incubated with rabbit anti-c-Fos antibody (sc-52: 1:1000, Santa Cruz, CA) overnight. Then, the sections were incubated for 30 min with biotinylated goat anti-rabbit IgG (Vector Laboratories, CA), diluted to 1:500. Following incubation, the sections were incubated with Alexa 594-labelled streptavidin, diluted to 1:500, for 30 min. The sections were mounted with mount medium (DAKO, CA). The images were acquired using a fluorescent microscope (Keyence, Osaka, Japan) or a confocal laser-scanning microscope (Fluoreview FV10i; Olympus, Osaka, Japan).

The numbers of c-Fos-IR cells, CTB-labelled cells, and both immunoreactive and CTB-labelled cells per section were counted from the fluorescence images. The cell count per section was averaged for all sections in each investigated nucleus of each animal.

### Statistical analysis

All data are presented as mean ± SEM. Student’s *t*-test was used for two-group comparisons. The statistical analysis of the locomotor activity, diurnal feeding pattern, the daily changes of BW and food intake were analyzed by two-way ANOVA followed by Tukey’s multiple range test. All statistical tests were two-tailed, with values of 0.05 being considered statistically significant.

## Result

### Distribution of c-Fos after refeeding, and GLP-1R positive neurons in DMH

The amount of food intake for 2 h in the refed rats was 3.46 ± 0.17 g. C-Fos expression was increased after refeeding in various feeding-related brain regions (Fig. 1G), including the SCN (Fig. 1A, D), DMH (Fig. 1B, E), and NTS (Fig. 1C, F). Also, GLP-1R immunostaining confirmed abundant GLP-1R expression in the entire area of the DMH (Fig. 1H–J), as well as PVN, VMH and ARC (Additional file 1: Fig. S3).

**Fig. 3:**
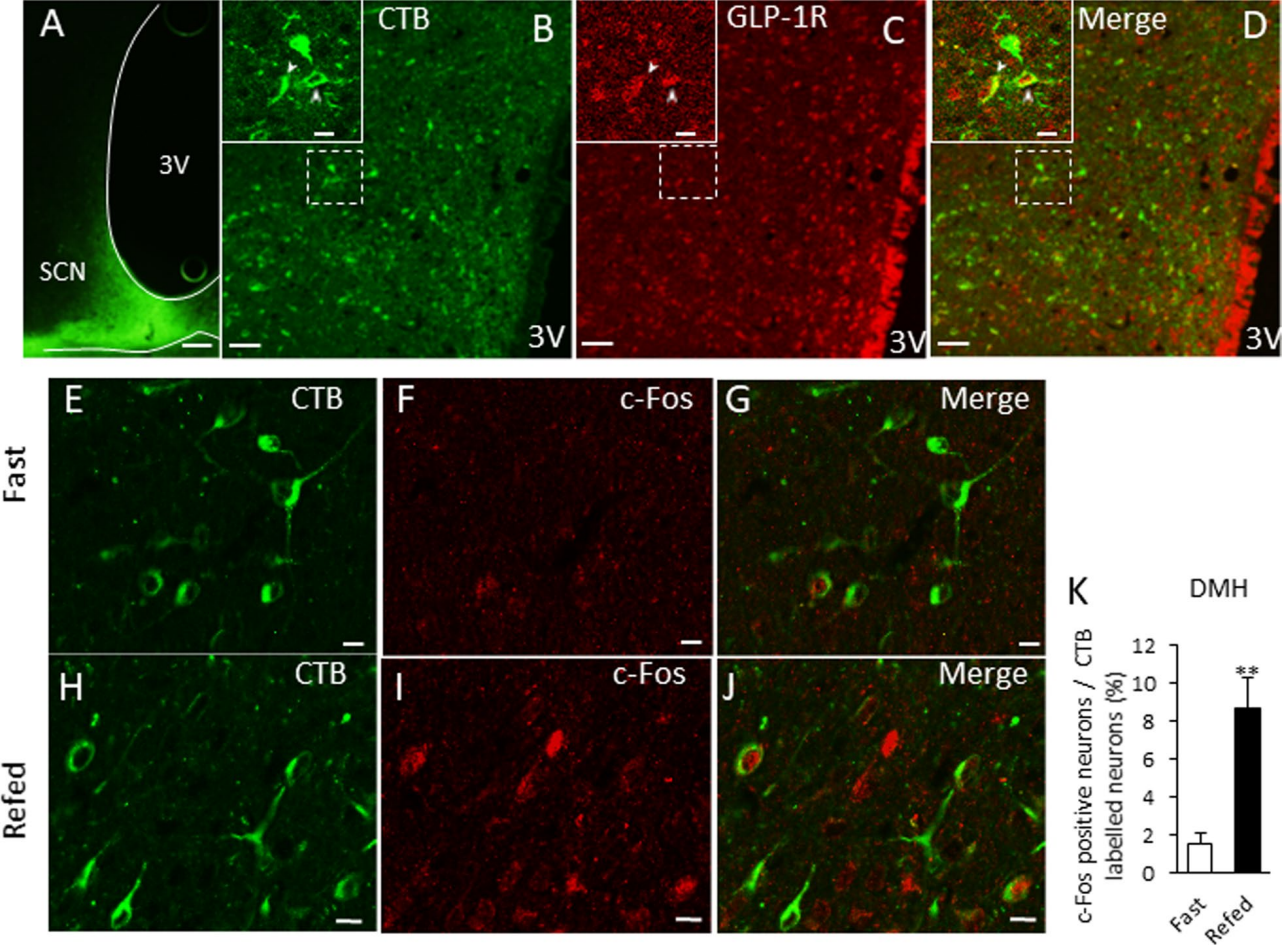
SCN projecting DMH neurons with GLP-1R expression increase c-Fos after feeding. **A** The injection site of the retrograde tracer, CTB in the SCN. Scale bar = 100 μm. **B** The distribution of CTB-positive neurons in the DMH. **C** The distribution of GLP-1R-positive neurons in the DMH. **D** A merged image of **B** and **C**. Scale bars = 50 μm. The enlarged images located in the left corner in the image of **B**–**D** indicate the image in the dotted white square in the respective image. Scale bars = 10 μm. Arrowheads indicate the neurons co-localizing CTB and GLP-1R. **E**–**J**: The distribution of CTB-positive neurons (**E**, **H**), c-Fos-positive neurons (**F**, **I**) and a merged image (**G**, **J**) in the DMH of a control fasting (**E**–**G**) or refeeding (**H**–**J**) rat. Scale bars in **E**–**J** indicate 10 μm. **K** The percentage of c-Fos-positive neurons among CTB-positive neurons in the DMH. (*n* = 5, 4) ** *P* < 0.01, Student *t*-test

### The deletion of GLP-1R-expressing neurons in the DMH induced hyperphagia and obesity, and disrupt diurnal feeding pattern

Exendin-4, and its synthetic version, exenatide 4, is a high-affinity agonist to GLP-1R [23]. Therefore, we injected Ex4-SAP into the bilateral DMH (Fig. 2A), where GLP-1R expression was observed. The Ex4-SAP-injected rats confirmed to have a low density of neurons in the DMH (Fig. 2B, C) with a decreased expression of GLP-1R mRNA. GLP-1R expression was decreased by approximately 90% (Fig. 2D). In addition to GLP-1R mRNA, GAD67 and CCKAR mRNA expressions were decreased by 20% (Fig. 2E, G). However, there were no changes in neuropeptide Y (NPY) and leptin receptor (LEPR) mRNA expression levels (Fig. 2F, H). Histochemical analysis revealed that the neurons with both GLP-1R and GAD67 positive neurons were frequently detected in the DMH (Fig. 2I–N). Twenty-five % of GLP-1R positive neurons were GAD67 positive, and 60% GAD67 positive neurons were GLP-1R positive (Table 2).

**Table 2:** Co-localization of GLP-1R and GAD67 in DMH

	Total GLP-1 neurons	Total GAD67 neurons	Total double positive neurons	% of GAD67 positive neurons in GLP-1R positive neurons	% of GLP-1R positive neurons in GAD67 positive neurons
Rat1	751	371	273	73.58	36.35
Rat2	1412	544	340	62.50	24.08
Rat3	790	293	148	50.51	18.73
Mean	984.33	402.67	253.67	62.20	26.39
SE	214.13	74.17	56.26	6.66	5.22

Fourteen days after Ex4-SAP injection, locomotor activity and food intake were measured for 24 h. The control rats showed significantly higher locomotor activity in the dark phase than in the light phase (Fig. 2O, P left). However, the Ex4-SAP-injected rats showed high locomotor activity throughout both phases with no significant difference in locomotor activity between dark and light phase (Fig. 2O, P left). The percentages of locomotor activity in dark and light phase were 67.3, 32.6% in B-SAP group, and 48.1, 51.9% in Ex4-SAP group, respectively (Fig. 2P right). In the control rats, the food intake was significantly higher in the dark phase than in the light phase (Fig. 2Q, R left), whereas there were no significant differences in the food intake between the light and dark phases in the Ex4-SAP-injected rats (Fig. 2Q, R left). The percentages of food intake in dark and light phase are 59.6, 40.4% in B-SAP group, and 52.7, 47.3% in Ex4-SAP group, respectively (Fig. 2R right).

To investigate the long-term effect of Ex4-SAP injection, food intake and BW were measured for 4 weeks after Ex4-SAP injection. Food intake was increased within 4 days after Ex4-SAP injection, and maximum food intake was detected at around 2 weeks after injection (Fig. 2S). The cumulative food intake of the Ex4-SAP-injected rats for 4 weeks was approximately twice that of the B-SAP-injected rats (Fig. 2T). Accompanying this, BW was also dramatically increased in the Ex4-SAP-injected rats (Fig. 2U). At 4 weeks after Ex4-SAP-injection, the difference in BW was approximately 100 g (Fig. 2U). The overall sizes of the animals were significantly different, as shown in Fig. 2V.

To examine the role of GLP-1R expressing neurons in the DMH, c-Fos expression levels in the SCN after fasting and refeeding were examined in rats that had their DMH injected with B- or Ex4-SAP. At 2 h after refeeding, there were no significant differences in food intake between the two groups (Fig. 2W). However, c-Fos expression after refeeding was decreased in the SCN of the Ex4-SAP-injected rats (Fig. 2X–Z). On the other hand, food intake after 6 to 12 and 12 to 24 h was decreased and increased, respectively in Ex4-SAP injected rats (Additional file 1: Fig. S4).

**Fig. 4:**
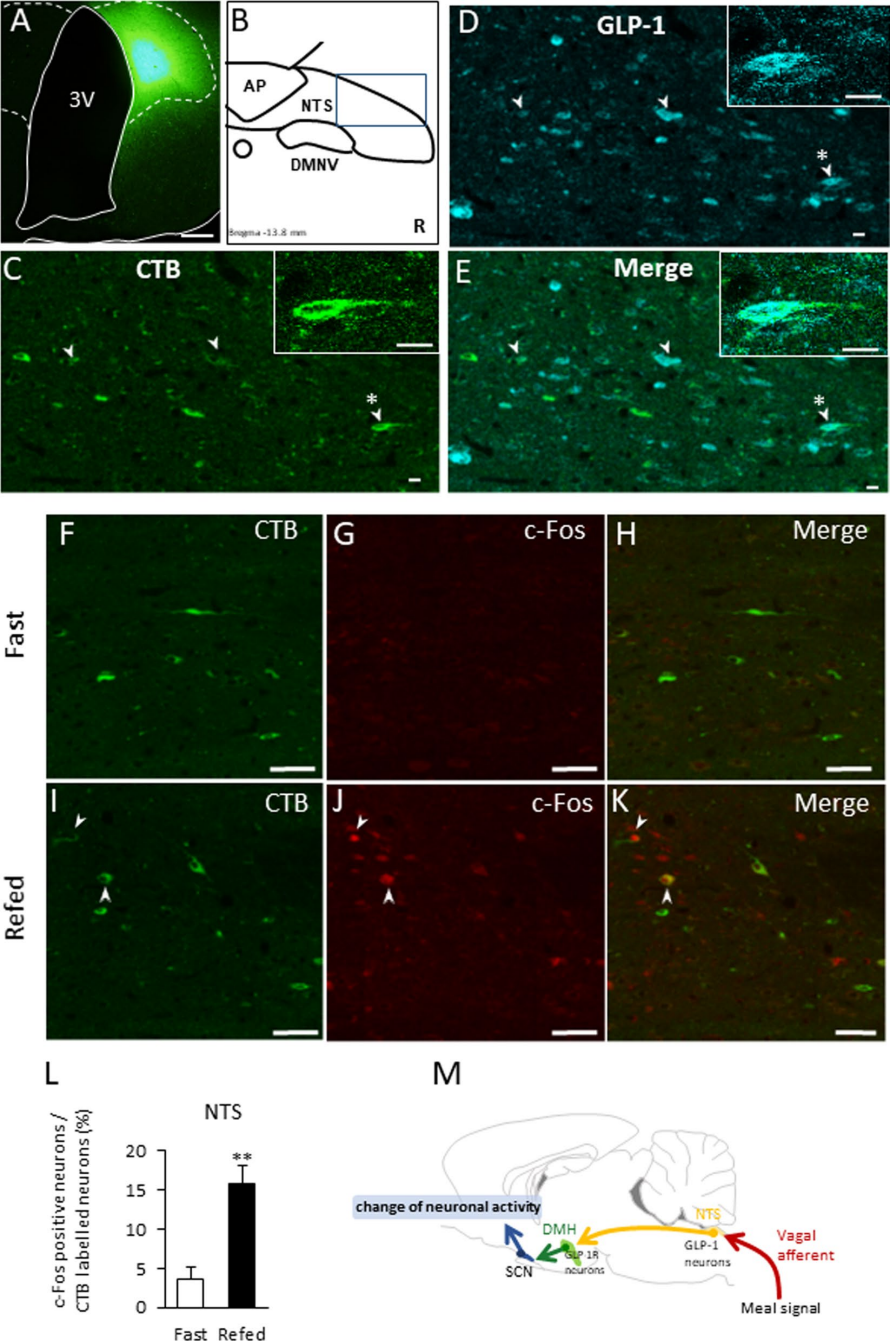
DMH projecting NTS neurons with GLP-1 expression increase c-Fos after feeding. **A** The injection site of the retrograde tracer, CTB in the DMH. Scale bar = 100 μm. **B** The scheme of dorsal vagal complex (AP: area postrema, NTS: nucleus tractus solitaries and DMNV: dorsal motor nucleus of the vagus) at − 13.8 mm from the bregma. **D**, **E** correspond with the blue square in **B** (lateral region of NTS). **D**: confocal image of GLP-1 immunoreactive neurons. **c** CTB-positive neurons in the NTS of rats with CTB injected into the DMH. **E** A merged image of **D** and **C**. Arrowheads indicate the double-positive neurons of GLP-1 and CTB. Scale bars = 10 μm. Enlarged images located in the right corner of image **D** and **E** correspond to the asterisk- (*) labelled neuron. **F**–**K** The distribution of CTB-positive neurons (**F**, **I**), c-Fos-positive neurons (**G**, **J**) and a merged image (**H**, **K**) in the NTS of a control fasting (**F**–**H**) or refeeding (**I**–**K**) rat. **l** The percentage of c-Fos-positive neurons among CTB-labelled neurons in the NTS after refeeding. (*n* = 4, 3). ** *P* < 0.01. **M** The scheme of the role of GLP-1R expressing neurons in the DMH. “meal” signal may be transmitted to SCN neurons and change the neural activities partially via NTS GLP-1 neurons and DMH GLP-1R expressing neurons

These results suggest that the GLP-1R-expressing neurons in the DMH may mediate the long-term satiety and change the activity of neurons in SCN after meal.

### Identification of projection from DMH GLP-1R-positive neurons to the SCN

Next, to clarify the neural signalling pathway from the DMH to the SCN, CTB was injected into the SCN (Fig. 3A). CTB-labelled neurons were abundantly distributed in the DMH (Fig. 3B), VMH, preoptic area (POA), BNST, arcuate nucleus (ARC), medial amygdala (MeA) and parabrachial nucleus (PBN). This result indicates the existence of projection from neurons in these nuclei, including DMH to SCN.

We next examined the projection from the GLP-1R-positive neurons in the DMH to the SCN (Fig. 3C, D), and found that 34% of the CTB-positive neurons in the DMH were GLP-1R-positive (Table 3). Conversely, 24% of the GLP-1R-positive neurons were CTB-positive (Table 3).

**Table 3:** Co-localization of CTB and GLP-1R in DMH

	Total CTB positive neurons	Total GLP-1R neurons	Total double positive neurons	% of CTB positive neurons in GLP-1R positive neurons	% of GLP-1R positive neurons in CTB positive neurons
Rat1	979	1158	302	26.08	30.85
Rat2	729	920	209	22.72	28.67
Rat3	753	1476	325	22.02	43.16
Mean	820.33	1184.67	278.67	23.61	34.23
SE	79.64	161.06	35.46	1.25	4.51

To identify the nuclei that are associated with the feeding signal, as well as the projection from the DMH to the SCN, c-Fos expression was measured in the brains of the SCN-CTB-injected rats after fasting and refeeding. Significant increases in c-Fos expression after refeeding in CTB-positive neurons were detected in the DMH (Fig. 3E–J) of the SCN-CTB-injected rats. Approximately 9% of the CTB-labelled neurons in the DMH were found to be c-Fos positive (Fig. 3K).

These histological data suggest that a substantial number of neurons projecting from the DMH to the SCN are GLP-1R-positive neurons and the possibility that a part of the projections from the DMH to the SCN influence the activities of neurons in the SCN in response to the meal.

### Identification of projection from NTS GLP-1 neurons to the DMH

To clarify the upstream neural pathway for the DMH-SCN projection, we performed GLP-1 immunostaining in the NTS-containing sections from rats with CTB injected into the DMH (Fig. 4A). CTB-labelled neurons were observed to be distributed in the NTS (Fig. 4B, C), PBN, locus coeruleus (LC), dorsal raphe (DR), MeA, PVN, ARC, anterior hypothalamic area (AHA), SCN, BNST and POA. GLP-1-immunoreactive neurons were found to be distributed in the lateral region of the NTS (Fig. 4D), and reticular nucleus. Approximately 33% of CTB-positive NTS neurons were identified as GLP-1-immunoreactive (Table 4), and 7.6% of GLP-1-immunoreactive neurons were CTB-positive. (Table 4). To identify the nuclei that are associated with feeding, as well as a projection to the DMH, c-Fos expression was measured in the brains of the DMH-CTB-injected rats after fasting and refeeding. Significant increases in c-Fos expression in CTB-positive neurons after refeeding were detected in the NTS (Fig. 4F–K) of DMH-CTB injected rats. Approximately, 15% of the CTB labelled neurons in the NTS were c-Fos positive (Fig. 4L). These data provide histological evidence that GLP-1 neurons in the NTS project to the DMH, and that a part of the projection from NTS to DMH may influence the activities of neurons in the SCN in response to feeding (Fig. 4M).

**Table 4:** Co-localization of CTB and GLP-1 in NTS

	Total CTB positive neurons	Total GLP-1 neurons	Total double positive neurons	% of CTB positive neurons in GLP-1 positive neurons	% of GLP-1 positive neurons in CTB positive neurons
Rat1	112	656	44	6.71	39.29
Rat2	86	468	33	7.05	38.37
Rat3	172	613	47	7.67	27.33
Rat4	202	615	55	8.94	27.23
Mean	588.00	143.00	44.75	7.59	33.05
SE	41.21	26.66	4.55	3.34	0.49

## Discussion

Recent studies have reported a close relationship between circadian rhythm and obesity. While the light and dark cycle is known to be a dominant zeitgeber for most organs, periodic food availability also represents a strong zeitgeber [2]. The vagal afferent-NTS neural system is important for conveying information on the feeding signal to the central nervous system [24]. Therefore, we consider that meal signals via the vagal afferent-NTS contribute to give some influence on activity of SCN neurons.

As a first step, we examined the c-Fos expression in the hypothalamus and brain stem after refeeding. Increases of c-Fos expression in the SCN after refeeding suggested that meal-evoked factors indeed changed the neural activities in the SCN.

Several mechanisms can be considered for the induction of neuronal activity in the SCN by refeeding. Such mechanisms should be triggered by factors that increase after eating. One such factor is humoral nutrients such as glucose and amino acids [25, 26] or humoral hormones [27, 28]. The ARC, which is known as the first-order nucleus of the feeding center, senses information from peripheral signals such as ghrelin, leptin [27], and insulin [28]. The importance of communication from the ARC to the SCN in maintaining the circadian rhythm has already been reported [6, 29]. Thus, the humoral nutrient/hormones → ARC → SCN neural pathway is considered to be one of the pathways that activate SCN neurons after refeeding.

Another possible neural pathway for refeeding-induced SCN activation is through gut hormone signals via the vagal afferent pathway, such as CCK, insulin, and GLP-1 [24, 30]. Because disruption of GLP-1R-expressing neurons in the DMH decreased c-Fos expression in the SCN after refeeding in the present study, we focused on the DMH mediated-neural pathway to the SCN after refeeding.

A possible upstream factor that regulates GLP-1R-positive neurons is neurons in the NTS, which is one of the brain sites where GLP-1-expressing neurons reside. Our experiment using CTB tracer also confirmed the projection from NTS GLP-1 positive neurons to DMH. Vrang et al. suggested that the neurons projecting from the NTS to the DMH are mainly PPG neurons [31], and Renner et al. suggested that GLP-1 neurons in the NTS activate DMH neurons via GLP-1R by conveying signals of satiety [32]. Previous articles have reported that the DMH play a key role as a food-entrainable oscillator in the feeding-mediated regulation of the circadian rhythm [21, 33].

Because there are very few reports regarding the contribution of DMH neurons on meal-oriented circadian rhythm regulation, we focused on the upstream of the DMH. We found that DMH neurons receive projections from various feeding-related brain regions, including the ARC, PVN, and NTS, which is consistent with the findings of previous studies [31, 34]. Among these nuclei, aside from the ARC, the NTS is also considered to be a first-order nucleus of the feeding center that receive feeding signals through the vagal afferent. There are several types of neurons in the NTS that contain neural peptides, such as GLP-1, POMC, CART, and PrRP [7]. The present study revealed that, in the NTS, 15% of DMH-projecting neurons were significantly activated after refeeding, and 33% of the DMH-projecting neurons were identified as GLP-1-positive. GLP-1 neurons in the NTS are closely related to feeding regulation affected by gut hormones. Intraperitoneal injection of the gut hormone CCK is known to activate GLP-1 neurons in the NTS [35]. Many other studies have reported the effect of GLP-1 in reducing food intake through the activation of neurons in the PVN [36, 37], lateral septum area [38], and reward-related nuclei, including the ventral tegmental area and nucleus accumbens [39, 40]. Renner et al. have reported that GLP-1-immunoreactive fibers and terminals topographically overlap with activated c-Fos-positive neurons in the DMH in refed rats [32].

In the current study, we found a marked distribution of GLP-1R in the DMH, which was also reported by Cork et al. [9] and Lee et al. [19]. Concerning with physiological role of neuron with GLP-1R expression in DMH, previous report by Lee et al., [19], which performed GLP-1R knock down (KD) in DMH, showed contradictory results from our present data which we deleted GLP-1R expressing neurons in DMH. Although BW was increased in both GLP-1R KD study and our deletion of GLP-1R expressing neurons, our data showed increase in food intake whereas not in study with GLP-1R KD in DMH. In addition, NPY mRNA expression in DMH was increased in GLP-1R KD, but showed no change in our rats with deletion of GLP-1R expressing neurons in DMH. Also, expression of CCK receptor and leptin receptor mRNA in DMH were not affected in GLP-1 KD rats, but CCK receptor mRNA were dramatically decreased in our rats with GLP-1R expressing neurons deleted in DMH. The underlying mechanism for these two contradicting results is unclear. Considering the fact that our Ex4-SAP injected rats showed dramatical hyperphagia and obesity (30% of BW increase), one possible mechanism could be the involvement of CCKR. The Ex4-SAP injected rats showed marked reduction of CCKR expression in DMH. CCKR in DMH is the key factor that regulate food intake. Zhu et al., reported that induction of CCKR in DMH of CCKR defected OLETF rats showed decrease of individual meal size with no effect on total amount of food intake and BW [41]. Therefore, decreased CCKR expression in DMH in Ex4-SAP injected rats may partially contribute to induction of hyperphagia and obesity. However, other factors may have been altered in our Ex4-SAP injected rats and therefore further studies are required.

Our present study showed that in Ex4-SAP injected rats, hyperphagia was observed in daily food intake at 28 days after the injection. The amount of refed food intake within 2 h after overnight fasting was not increased but food intake of 6 to 12 h was decreased, and 12 to 24 h was increased. These data suggest that GLP-1R expressing neurons are related to long-term feeding regulation, through changes in locomotor activity and feeding rhythm via SCN neuronal activity, rather than short term effect or acute phase changes in satiety cognition.

In animals that anticipate scheduled food, c-Fos expression in the SCN is reported to be low and neuronal activity in the DMH to be high [42]. Also, it has been demonstrated that the inhibition of SCN neuronal activity is a consequence of activated GABA-expressing neurons in the DMH that project to the SCN [42]. These reports indicate the importance of inhibitory input from the DMH to the SCN for food-entrainable circadian rhythm.

However, in this study, refeeding induced c-Fos expression in both the SCN and DMH, and c-fos expression was decreased in the SCN of the rats with disrupted GLP-1R-positive neurons in the DMH. These data suggest that the SCN receives activatory inputs from DMH neurons after refeeding. It is reported that 16% of GLP-1R expressing neurons are glutamatergic neurons [19]. On the other hand, 25–35% of GLP-1R positive neurons are GABAergic [19].

The mRNA expression of GAD67, which are critical enzyme for synthesizing GABA, was dramatically decreased after deletion of GLP-1R positive neurons in the current study. Histological analysis also found that 60% of GAD67 positive neurons are GLP-1R positive neurons, and 25% of GLP-1R positive neurons are GAD67 positive neurons in DMH which is consistent to the previous article [19]. The contribution of this GAD67 reduction remains unclear. In this study, together with hyperphagia, increase of locomotor activities were confirmed after Ex4-SAP injection. However, excitotoxic lesion of DMH is reported to reduce food intake [43] circadian rhythms of wakefulness, feeding and locomotor activity [44]. Therefore, the effects of specific deletion of GLP-1R expressing neurons in the DMH are different from lesioning of DMH. In this study, we confirmed that expression of GAD67 mRNA in DMH was reduced in Ex4-SAP injected rats and therefore the GABA signals should have been reduced in these rats. Considering from previous reports and our data, it is possible to consider that reduction of both GABAergic and glutamatergic neurons in DMH GLP-1R expressing neurons may have altered the overall neural activities of SCN in our Ex4-SAP injected rats. Our present data indicate that disturbance of overall excitatory/inhibitory input from DMH GLP-1R expressing neurons may alter circadian rhythm-based food intake and body weight regulation. This interpretation supports our suggestion that GLP-1R expressing neurons in DMH are related to long-term feeding regulation, through changes in locomotor activity and feeding rhythm via SCN neuronal activity, rather than acute phasic satiety cognition. However, further studies are required to clarify this in the future.

## Conclusion

In the current study, we showed that the disruption of GLP-1R expressing neurons in the DMH eliminates diurnal feeding pattern, and induces both hyperphagia and severe obesity. Additionally, we showed that feeding activated SCN neurons, partially via the NTS-DMH neural pathway.

The present study indicates the “meal” signal may be transmitted to SCN neurons and change the neural activities via DMH GLP-1R expressing neurons.

## Supplementary Information

**Additional file 1**. **Supplemental Fig. 1.** Absorption test for GLP-1R antibody. **Supplemental Fig. 2.** Western blotting using GLP-1R antibody. **Supplemental Fig. 3.** GLP-1R distribution in the PVN, VMH and ARC. **Supplemental Fig. 4.** Food intake after refeeding.

## Data Availability

The datasets used and analyzed during the current study available from the corresponding author on reasonable request.

## Abbreviations

AHA: Anterior hypothalamic area
ANOVA: Analysis of variance
ARC: Arcuate nucleus
BAT: Brown adipose tissue
B-SAP: Blank-saporin
BNST: Bed nucleus of the stria terminalis
BSA: Bovine serum albumin
BW: Body weight
CART: Cocaine- and amphetamine-regulated transcript
CCK: Cholecystokinin
CCKAR: Cholecystokinin receptors
CTB: Cholera toxin subunit B
DMH: Dorsomedial hypothalamic nucleus
DR: Dorsal raphe
Ex4: Exenatide 4
Ex4-SAP: Exenatide 4-saporin
GABA: γ-Aminobutyric acid
GAD: Glutamic acid decarboxylase
GLP-1: Glucagon-like peptide-1
GLP-1R: Glucagon-like peptide-1 receptors
KD: Knock down
LC: Locus ceruleus
LEPR: Leptin receptors
MeA: Medial amygdala
NGS: Normal goat serum
NPY: Neuropeptide Y
NTS: Nucleus tractus solitarius
PAG: Periaqueductal gray
PBN: Parabrachial nucleus
PFA: Paraformaldehyde
PrRP: Prolactin-releasing peptide
POA: Preoptic area
POMC: Proopiomelanocortin
PPG: Preproglucagon
PVN: Paraventricular hypothalamic nucleus
PVT: Paraventricular thalamic nucleus
SAP: Saporin
SCN: Sprachiasmatic nucleus
VMH: Ventromedial hypothlamic nucleus
ZT: Zeitgeber time

## References

[1] Challet E (2013). Circadian clocks, food intake, and metabolism. Prog Mol Biol Transl Sci.

[2] Wu T, Jin Y, Kato H, Fu Z (2008). Light and food signals cooperate to entrain the rat pineal circadian system. J Neurosci Res.

[3] Mouret J, Coindet J, Debilly G, Chouvet G (1978). Suprachiasmatic nuclei lesions in the rat: alterations in sleep circadian rhythms. Electroencephalogr Clin Neurophysiol.

[4] Turek FW, Joshu C, Kohsaka A, Lin E, Ivanova G, McDearmon E (2005). Obesity and metabolic syndrome in circadian Clock mutant mice. Science.

[5] Morton GJ, Cummings DE, Baskin DG, Barsh GS, Schwartz MW (2006). Central nervous system control of food intake and body weight. Nature.

[6] Li AJ, Wiater MF, Oostrom MT, Smith BR, Wang Q, Dinh TT (2012). Leptin-sensitive neurons in the arcuate nuclei contribute to endogenous feeding rhythms. Am J Physiol Regul Integr Comp Physiol.

[7] Cone RD (2005). Anatomy and regulation of the central melanocortin system. Nat Neurosci.

[8] Crane J, McGowan B (2016). The GLP-1 agonist, liraglutide, as a pharmacotherapy for obesity. Ther Adv Chronic Dis.

[9] Cork SC, Richards JE, Holt MK, Gribble FM, Reimann F, Trapp S (2015). Distribution and characterisation of Glucagon-like peptide-1 receptor expressing cells in the mouse brain. Mol Metab.

[10] Llewellyn-Smith IJ, Reimann F, Gribble FM, Trapp S (2011). Preproglucagon neurons project widely to autonomic control areas in the mouse brain. Neuroscience.

[11] Williams DL, Lilly NA, Edwards IJ, Yao P, Richards JE, Trapp S (2011). GLP-1 action in the mouse bed nucleus of the stria terminalis. Neuroscience.

[12] Gaykema RP, Newmyer BA, Ottolini M, Raje V, Warthen DM, Lambeth PS, et al. Activation of murine pre-proglucagon-producing neurons reduces food intake and body weight. J Clin Invest. 2017;127:1031–45. 10.1172/JCI81335. 10.1172/JCI81335 PMC5330725 28218622

[13] Barrera JG, Jones KR, Herman JP, D'Alessio DA, Woods SC, Seeley RJ (2011). Hyperphagia and increased fat accumulation in two models of chronic CNS glucagon-like peptide-1 loss of function. J Neurosci.

[14] Holt MK, Richards JE, Cook DR, Brierley DI, Williams DL, Reimann F (2019). Preproglucagon neurons in the nucleus of the solitary tract are the main source of brain GLP-1, mediate stress-induced hypophagia, and limit unusually large intakes of food. Diabetes.

[15] Barrera JG, Sandoval DA, D'Alessio DA, Seeley RJ (2011). GLP-1 and energy balance: an integrated model of short-term and long-term control. Nat Rev Endocrinol.

[16] Bi S, Kim YJ, Zheng F (2012). Dorsomedial hypothalamic NPY and energy balance control. Neuropeptides.

[17] Chaar LJ, Coelho A, Silva NM, Festuccia WL, Antunes VR. High-fat diet-induced hypertension and autonomic imbalance are associated with an upregulation of CART in the dorsomedial hypothalamus of mice. Physiol Rep. 2016;4:e12811. 10.14814/phy2.12811. 10.14814/phy2.12811 PMC4908489 27273815

[18] de Noronha SR, Campos GV, Abreu AR, de Souza AA, Chianca DA, de Menezes RC (2017). High fat diet induced-obesity facilitates anxiety-like behaviors due to GABAergic impairment within the dorsomedial hypothalamus in rats. Behav Brain Res.

[19] Lee SJ, Sanchez-Watts G, Krieger JP, Pignalosa A, Norell PN, Cortella A (2018). Loss of dorsomedial hypothalamic GLP-1 signaling reduces BAT thermogenesis and increases adiposity. Mol Metab.

[20] Landry GJ, Kent BA, Patton DF, Jaholkowski M, Marchant EG, Mistlberger RE (2011). Evidence for time-of-day dependent effect of neurotoxic dorsomedial hypothalamic lesions on food anticipatory circadian rhythms in rats. PLoS ONE.

[21] Mieda M, Williams SC, Richardson JA, Tanaka K, Yanagisawa M (2006). The dorsomedial hypothalamic nucleus as a putative food-entrainable circadian pacemaker. Proc Natl Acad Sci USA.

[22] Wiley RG, Kline RH (2000). Neuronal lesioning with axonally transported toxins. J Neurosci Methods.

[23] Göke R, Fehmann HC, Linn T, Schmidt H, Krause M, Eng J, et al. Exendin-4 is a high potency agonist and truncated exendin-(9–39)-amide an antagonist at the glucagon-like peptide 1-(7–36)-amide receptor of insulin-secreting beta-cells. J Biol Chem. 1993;268:19650–5. 8396143

[24] Schwartz GJ (2006). Integrative capacity of the caudal brainstem in the control of food intake. Philos Trans R Soc Lond B Biol Sci.

[25] Froy O (2007). The relationship between nutrition and circadian rhythms in mammals. Front Neuroendocrinol.

[26] Wu T, Yao C, Huang L, Mao Y, Zhang W, Jiang J (2015). Nutrients and circadian rhythms in mammals. J Nutr Sci Vitaminol (Tokyo).

[27] Kohno D, Gao HZ, Muroya S, Kikuyama S, Yada T (2003). Ghrelin directly interacts with neuropeptide-Y-containing neurons in the rat arcuate nucleus: Ca^2+^ signaling via protein kinase A and N-type channel-dependent mechanisms and cross-talk with leptin and orexin. Diabetes.

[28] Maejima Y, Kohno D, Iwasaki Y, Yada T. Insulin suppresses ghrelin-induced calcium signaling in neuropeptide Y neurons of the hypothalamic arcuate nucleus. Aging (Albany NY). 2011;3:1092–7. 10.18632/aging.100400. 10.18632/aging.100400 PMC3249454 22081645

[29] Yi CX, van der Vliet J, Dai J, Yin G, Ru L, Buijs RM (2006). Ventromedial arcuate nucleus communicates peripheral metabolic information to the suprachiasmatic nucleus. Endocrinology.

[30] Suzuki K, Simpson KA, Minnion JS, Shillito JC, Bloom SR (2010). The role of gut hormones and the hypothalamus in appetite regulation. Endocr J.

[31] Vrang N, Hansen M, Larsen PJ, Tang-Christensen M (2007). Tang-Christensen, Characterization of brainstem preproglucagon projections to the paraventricular and dorsomedial hypothalamic nuclei. Brain Res.

[32] Renner E, Puskás N, Dobolyi A, Palkovits M (2012). Glucagon-like peptide-1 of brainstem origin activates dorsomedial hypothalamic neurons in satiated rats. Peptides.

[33] Gooley JJ, Schomer A, Saper CB (2006). The dorsomedial hypothalamic nucleus is critical for the expression of food-entrainable circadian rhythms. Nat Neurosci.

[34] Dai J, Van Der Vliet J, Swaab DF, Buijs RM (1998). Postmortem anterograde tracing of intrahypothalamic projections of the human dorsomedial nucleus of the hypothalamus. J Comp Neurol.

[35] Maniscalco JW, Rinaman L (2013). Overnight food deprivation markedly attenuates hindbrain noradrenergic, glucagon-like peptide-1, and hypothalamic neural responses to exogenous cholecystokinin in male rats. Physiol Behav.

[36] Larsen PJ, Tang-Christensen M, Jessop DS (1997). Central administration of glucagon-like peptide-1 activates hypothalamic neuroendocrine neurons in the rat. Endocrinology.

[37] McMahon LR, Wellman PJ (1998). PVN infusion of GLP-1-(7–36) amide suppresses feeding but does not induce aversion or alter locomotion in rats. Am J Physiol.

[38] Terrill SJ, Jackson CM, Greene HE, Lilly N, Maske CB, Vallejo S (2016). Role of lateral septum glucagon-like peptide 1 receptors in food intake. Am J Physiol Regul Integr Comp Physiol.

[39] Alhadeff AL, Rupprecht LE, Hayes MR (2012). GLP-1 neurons in the nucleus of the solitary tract project directly to the ventral tegmental area and nucleus accumbens to control for food intake. Endocrinology.

[40] Dossat AM, Lilly N, Kay K, Williams DL (2011). Glucagon-like peptide 1 receptors in nucleus accumbens affect food intake. J Neurosci.

[41] Zhu G, Yan J, Smith WW, Moran TH, Bi S (2012). Roles of dorsomedial hypothalamic cholecystokinin signaling in the controls of meal patterns and glucose homeostasis. Physiol Behav.

[42] Acosta-Galvan G, Yi CX, van der Vliet J, Jhamandas JH, Panula P, Angeles-Castellanos M (2011). Interaction between hypothalamic dorsomedial nucleus and the suprachiasmatic nucleus determines intensity of food anticipatory behavior. Proc Natl Acad Sci USA.

[43] Bernardis LL, Bellinger LL (1998). The dorsomedial hypothalamic nucleus revisited: 1998 update. Proc Soc Exp Biol Med.

[44] Chou TC, Scammell TE, Gooley JJ, Gaus SE, Saper CB, Lu J (2003). Critical role of dorsomedial hypothalamic nucleus in a wide range of behavioral circadian rhythms. J Neurosci.

