# Supplementary material for: The deletion of glucagon-like peptide-1 receptors expressing neurons in the dorsomedial hypothalamic nucleus disrupts the diurnal feeding pattern and induces hyperphagia and obesity
Source: Nutr Metab (Lond). 2021 Jun 7;18:58. doi: 10.1186/s12986-021-00582-z (PMC8186199; doi:10.1186/s12986-021-00582-z)
Supplement: Supplementary file 1 — Additional file 1. Supplemental Fig. 1. Absorption test for GLP-1R antibody. Supplemental Fig. 2. Western blotting using GLP-1R antibody. Supplemental Fig. 3. GLP-1R distribution in the PVN, VMH and ARC. Supplemental Fig. 4. Food intake after refeeding. [file 12986_2021_582_MOESM1_ESM.docx]

**The deletion of glucagon-like peptide-1 receptors expressing neurons in the dorsomedial hypothalamic nucleus disrupts the diurnal feeding pattern and induces hyperphagia and obesity**


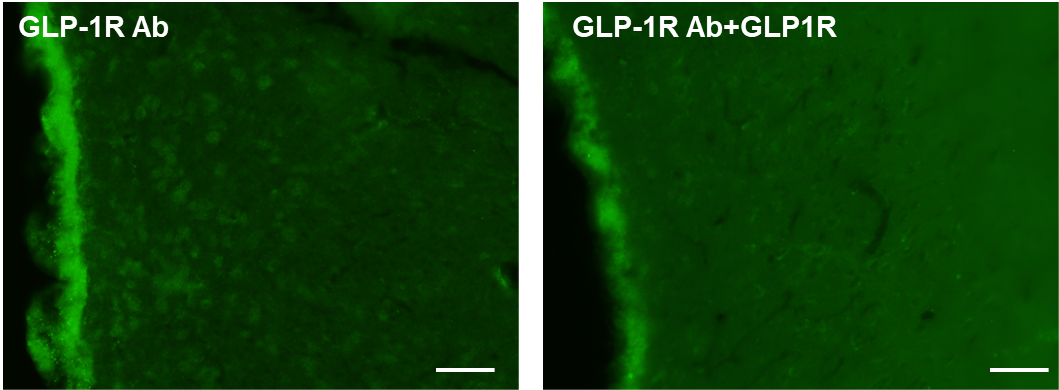


Supplemental Fig.1 Absorption test for GLP-1R antibody

Rabbit anti-GLP-1R antibody was pre-incubated with recombinant rat GLP-1R (MBS966507, MyBioSource, CA) for overnight (Molecular ratio, antibody: GLP-1R = 1: 20). The staining procedure was same as described in the methods in the manuscript. The absorption test showed that immunoreactivity was completely abolished from all sections which were incubated with a pre-absorbed antibody with rat GLP-1R.

Left: The fluorescence image of DMH section incubated with GLP-1R antibody (ThermoFisher :PA5-33591).

Right: The fluorescence image of DMH section incubated with GLP-1R antibody, which were preincubated with rat GLP-1R (M Biosource, MBS966507).

Scale bars = 100 μm.


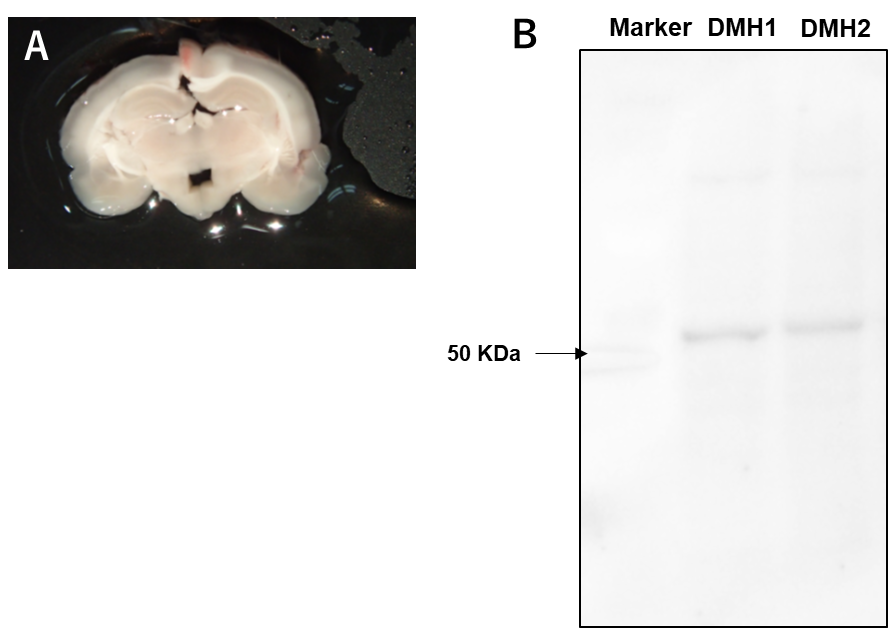


Supplemental Fig.2 Western blotting using GLP-1R antibody

A: Representative image showing DMH dissection for PCR and western blotting.

B: Western blot analysis of GLP1-R with extracts from rat DMH using GLP-1R Polyclonal Antibody (ThermoFisher: PA5-33591) at a dilution of 1:500. A HRP Goat Anti-Rabbit IgG (H+L) secondary antibody was used at a dilution of 1:1,000. Lysates: 50 µg per lane. Blocking buffer: 5% skim milk in PBS containing 0.025% tween 20. Marker: BIO RAD precision plus protein dual color standard, Cat#1610374

This antibody clearly detected 53 kD protein from rat DMH lysate.


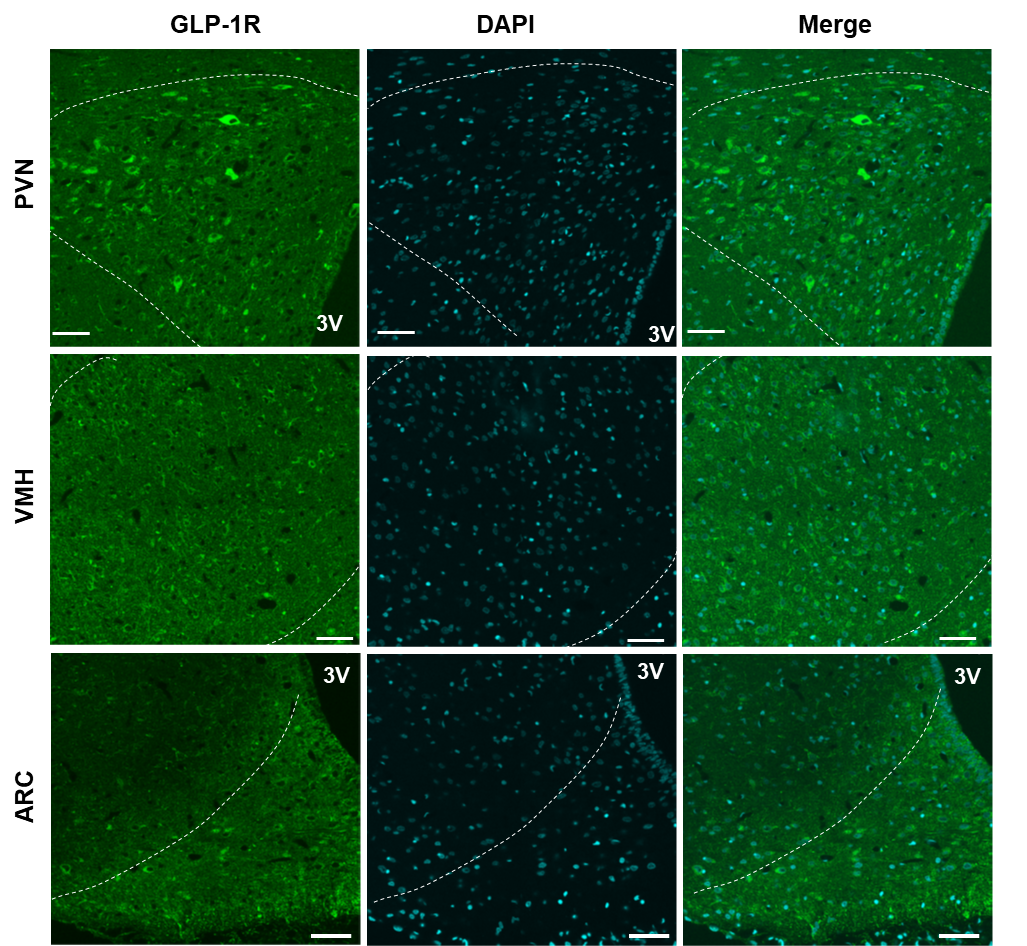


Supplemental Fig.3 GLP-1R distribution in the PVN, VMH and ARC.

3V: 3^rd^ ventricle. Scale bars = 50 μm.


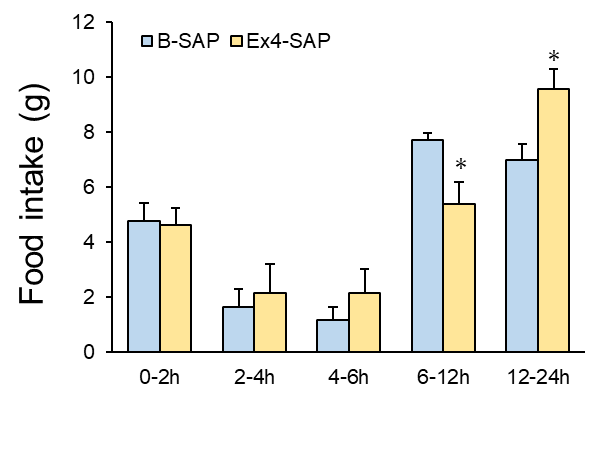


Supplemental Fig. 4 Food intake after refeeding.

At 29 days after B-SAP or Ex4-SAP injection, food was deprived at ZT10. Then, after 16 h of fasting, food was administered to the animals at ZT2, and food intake was measured for 0-2 h, 2-4 h, 4-6 h and 6-12h and 12-24 h. n = 4, 3. *P < 0.05. t-test.
